# Bullying in Nursing Students: A Cross-Sectional Study

**DOI:** 10.3390/ijerph21111431

**Published:** 2024-10-29

**Authors:** Lidia Fernández-Gutiérrez, Maria-Pilar Mosteiro-Díaz, Elisabete Borges, Sara Franco-Correia

**Affiliations:** 1 Health Service of the Principality of Asturias, 33006 Oviedo, Spain; lidia1791@hotmail.com; 2 Research Group INEUROPA and Research Group HWOPI—Health Work, Department of Medicine, Nursing Area, University of Oviedo, 33006 Oviedo, Spain; 3 Research Group HWOPI—Health Work, Nursing School of Porto, 4200-450 Porto, Portugal; elisabete@esenf.pt; 4 Department of Medicine, Nursing Area, University of Oviedo, 33006 Oviedo, Spain; sara.fr.correia@gmail.com

**Keywords:** bullying, nursing students, mental health

## Abstract

The presence of violence between coworkers in nursing settings is a common fact that has been documented over time by using different terms to define the violence suffered by nurses and nursing students, although in recent years it has been agreed that the term “bullying” covers them all. This study aims to determine the prevalence and characteristics of bullying in nursing students, and to describe the association between the prevalence of bullying and socio-demographic attributes. A descriptive, observational, quantitative, and cross-sectional study was carried out of nursing students between 2017 and 2020. Socio-demographic variables were collected. Bullying was assessed through the NAQ-R questionnaire. Data were analyzed using the R statistical program. A total of 411 nursing students were included. A prevalence of perceived bullying of 92% was detected. The main negative attitudes identified were mandated tasks that are below the level of competence (66.3%), changes in tasks for other more trivial or unpleasant ones (60%), having opinions that are ignored (52%), and being excessively supervised (49%). The prevalence of bullying in nursing students at the University of Oviedo exceeds 90%, which suggests that interventions to solve the problem of bullying are urgent at the institutional, political, and organizational levels.

## 1. Introduction

Violence framed in the nursing profession is not a new topic. The phenomenon of bullying and its consequences have been studied in depth over the years, defined by different terms that vary depending on the country [1]. In English-speaking countries and others like Turkey, the most frequently used term for violence between coworkers is “bullying” [2]. On the other hand, in German-speaking countries and the Netherlands, the term “mobbing” is used more [3].

There are several definitions of bullying depending on the field, specifically in the field of nursing. The American Nurse Association accepted the definition of bullying that defines it as a form of lateral, verbal or non-verbal, violence, in which acts of harassment are carried out by a person with a higher level of authority or by peers of the same level [4].

Bullying behaviours are damaging to nurses as well as those aspiring to become members of the largest healthcare profession [5]. Many new graduate nurses in their first year of employment, and also nursing students during their clinical rotation, experience issues at work such as violence, bullying, and stress [6]. It has been described that nurses and final-year nursing students experience challenges such as occupational stress, workplace violence, and bullying while adjusting to their new roles and transitioning to clinical practice [7,8]. This phenomenon has physical, psychological, emotional, professional, and organizational consequences [9,10,11,12,13]. With regard to the effects of bullying, in a qualitative phenomenological descriptive study of nursing students, participants reported that when faced with bullying, they experienced a loss of confidence, stress, and poor learning outcomes [14].

Bullying is a particularly worrying phenomenon, both because of the consequences it has and due to the high prevalence found in studies, of around 50–60% [15,16,17,18], rising in some cases to 80–90% [19,20]. Researchers are seeking to understand the bullying phenomenon in nursing students. There is an urgent need to find ways to intervene to mitigate the potential effects of exposure to bullying and prevent irreversible damage to the next generation of nurses [5]. It is clear that nursing students are adversely affected by bullying behaviours. 

This study aims to determine the prevalence and characteristics of bullying among Spanish nursing students during clinical practices, and to describe the association between the prevalence of bullying and socio-demographic characteristics.

## 2. Materials and Methods

### 2.1. Design

A descriptive, cross-sectional study of nursing students at a Spanish University was designed. Authors adhered to the STROBE checklist for observational research [21]. Data collection was performed between 2017 and 2020.

### 2.2. Participants

In Spain, nursing studies are divided into four academic courses, each lasting one year, with a total of 240 ECTS credits. At the selected university, clinical nursing practices begin in the second semester of the first year and are distributed transversally throughout the rest of the program. All nursing students from the 2017 to 2020 courses of the Nursing Degree at the University of Oviedo were included as the population of this study. It was only intended to evaluate the students once during their studies, after they had already had some clinical rotation, so data collection was distributed as follows: in the 2017–2018 academic year, 4th grade students were surveyed; in the 2018–2019 academic year, 2nd, 3rd, and 4th grade students were surveyed; in the 2019–2020 academic year, 2nd year students were surveyed. Inclusion criteria were defined as follows: being an undergraduate nursing student enrolled in the selected university, who was in the classroom at the time of data collection, had agreed to participate in the study, and had completed at least 80% of the questionnaires. A sample of 411 Spanish nursing students was obtained.

### 2.3. Data Collection Procedure

Once permission had been obtained from the Research Ethics Committee of the Principality of Asturias, as well as authorization from the respective directorates of the university centers, the students were contacted. Through a group discussion at the beginning of the academic year and during school hours, the study was presented, asking for the students’ collaboration and agreement to participate in the study, voluntarily, anonymously, and while maintaining confidentiality. The questionnaires were then delivered personally and collected after they had been completed. Clarification was given regarding the content of the questionnaires: it was reported that the NAQ-R referred to the time they spent practicing in the clinical setting. All questionnaires were identified with consecutive numbers, starting from 1, and they were also identified according to the center to which the student belonged.

### 2.4. Measures

The measurements included demographics and bullying.

#### 2.4.1. Socio-Demographic Data

Participants were asked to provide socio-demographic information: their age, gender, marital status, and academic year.

#### 2.4.2. Bullying

The Negative Acts Questionnaire-Revised (NAQ-R) [22] was used to evaluate whether the students were victims of negative attitudes from peers or superiors and to assess the prevalence of bullying. Cronbach’s alpha for the NAQ-R was 0.90 [22]. The Spanish version of the NAQ-R [23], which includes 22 items and an additional specific question on sexual harassment, was chosen as it is considered a prevalent and important issue nowadays. Additionally, as described in the original NAQ, a definition of harassment is introduced, and a question is asked regarding whether respondents have experienced this problem at work.

The NAQ-R also measures the frequency of exposure to negative attitudes in the last six months on a Likert scale ranging from 1 to 5. This is further evaluated through three factors (psychological harassment, work-related harassment, and physical abuse), with the sum of all the items yielding a minimum score of 24 and a maximum of 120.

When evaluating the questionnaire, an individual is considered a victim of bullying when they answer something other than “never” to two or more questions and/or answer affirmatively to the last question about self-perceived bullying. In addition, the three additional factors can be evaluated jointly or independently: work-related harassment—composed of the sum of items 1, 3, 14, 16, 18, 19, and 21 (minimum 1—maximum 35); psychological harassment—composed of the sum of items 2, 4, 5, 6, 7, 10, 11, 12, 13, 15, 17, and 20 (minimum 1—maximum 60); and physical abuse—composed of the sum of items 8, 9, and 22 (minimum 1—maximum 15) [22].

After collecting the data, statistical analysis was carried out. To minimize errors, the database was analyzed by two people independently. Statistical analysis was subsequently carried out with the R Program (R Development Core Team) (GNU Project, New Zealand), version 3.6.3. (2020). For the descriptive analysis of both socio-demographic variables and the NAQ-R, the following analysis was carried out to study the distribution of relative and absolute frequencies for qualitative variables, and dispersion measures for quantitative variables. To analyze the differences in the distribution of the qualitative variables in both groups, the Pearson chi-square test or Fisher’s exact test was used, depending on whether the hypothesis of normality was verified or not. The differences in quantitative variables between the two groups were assessed using Student’s *t*-test for independent samples. To analyze the factors associated with suffering from bullying, univariate and multivariate logistic regression models were built. The level of statistical significance used was 0.05.

### 2.5. Ethics

Ethical committee approval was obtained from the Ethical Committee of Research of the Principality of Asturias (CeiPA 38/18). Participants were informed about confidentiality and anonymity. Participation was voluntary. Before the data collection, participants were given written information regarding the study, and they were asked to provide informed consent. Legal and ethical aspects were considered throughout the research process.

## 3. Results

### 3.1. Sample Description

A response rate of 100% was obtained, with all of the 411 questionnaires delivered being collected. The mean age was 22.42 years, with a range of 18–49 years and a standard deviation of 5.05. The sample was mainly composed of women (84.9%), single/divorced respondents (89.8%), and those studying at the main center (61.6%). Socio-demographic results are shown in Table 1.

### 3.2. Prevalence of Bullying

To view a participant as a victim of bullying, the response to two or more items must be something other than “never” and/or an affirmative answer must be given to the last question of the questionnaire: “Have you been harassed at work?” Based on this, we found that 378 of our participants fulfilled one or both of these premises, which implies a prevalence of perceived bullying of 92%. Responding affirmatively to the last item of the questionnaire, regardless of the others, is synonymous with suffering bullying. According to this premise, 17.5% of our participants had suffered bullying, without having to count the other items, as shown in Table 2.

### 3.3. Prevalence of Negative Attitudes

The following table (Table 3) shows the prevalence of negative attitudes reported by respondents. As the most prevalent behaviours we find those referring to tasks (orders to carry out work due to a lower level of competence, changes in tasks of responsibility for more trivial ones, excessive supervision, or exposure to excessive workload).

### 3.4. NAQ-R Factors and Total NAQ-R Score

The three factors were analyzed independently, and it was verified that the highest score was obtained in the psychological harassment factor, with 50% of the respondents having an average score above 20 in this factor (where the maximum score is 35). The results of the three factors and the total score are reflected in Table 4.

### 3.5. Correlational Analysis

Associations between socio-demographic variables and bullying were studied through statistical analysis, searching for possible relationships between age, sex, marital status, academic year, and center. No statistically significant associations among any of these variables were detected. 

## 4. Discussion

In analyzing the literature on the phenomenon of bullying, we found similarities and differences in our results.

Among the participants in this study, 84.91% were women. This is in line with the National Institute of Statistics, which has recorded that the percentage of collegiate women in nursing in Spain is 84.15% [24]. Furthermore, our results concur, in general, with the distribution by gender found in other studies that have analyzed bullying among nursing students [25,26,27,28,29]. Considering these data, as well as what we know through history, it is evident that, today, nursing is still a profession in which most of its members are women, which is why data from our study are similar to others in regard to distribution by gender. 

We also found authors whose findings were similar regarding the proportion of students per academic course [12,30].

With regard to the prevalence of bullying, 92% of our participants met the criteria to be considered victims of bullying. The results of our study are along the same lines as those found in other investigations. In general, the prevalence of bullying experienced by nursing students is high, exceeding 70% in most cases [11,26,27,28,31,32,33,34]. Other studies find prevalences of around 50% [12,25,29,35,36,37,38,39]. It is important to point out that the results will also vary depending on the methodology employed and the tools used to collect the data.

In analyzing the responses obtained from the NAQ-R questionnaire, we found that the most frequent acts of harassment experienced by students were as follows: mandates to perform tasks that were due to a decrease in their level of competence (66.3%); changing from responsible tasks to more trivial or unpleasant ones (59.9%); opinions or points of view being ignored (51.6%); excessive supervision at work (48.9%); exposure to an excessive workload (47.3%); and being ignored or receiving a hostile reaction when approaching someone (38.5%). In relation to this aspect, it is important to clarify that in the context of the Spanish Healthcare System, there is a figure within the nursing hierarchy—the nursing assistant—who, due to the professional training and educational level, performs certain functions and tasks assigned in the patient care process, which are delegated and supervised by nurses. This practice is normalized in this context.

Regarding the results on this specific topic, we found disparity comparing our data to other studies. Clarke et al. [27] found that the most prevalent act of harassment was to have felt that the efforts that students carried out were undervalued (60%), followed by negative comments about becoming a nurse (45.25%). These results are in line with those found in the study of Reem-Mabrouk [26], in which what stands out most is that the efforts of students have barely been considered (83%) and they have received negative comments about becoming a nurse (82%). In our case, these last actions are not the worst suffered by our students because we obtained a prevalence of 18.8% in relation to negative comments about becoming a nurse and 21% in relation to feeling that their work was undervalued or excessively criticized.

The results of another study show that students have been ignored and excluded (29%), as well as experiencing destructive criticism (29%) and seeing their efforts undervalued (28%) [36]. Even though in our case the prevalence is lower, feeling ignored or excluded is also a significant problem that impacts students, just as it does in our study.

Abdelaziz and Abu-Snieneh [37] used a methodology similar to that employed in this study, with a more summarized version of the NAQ, and obtained results that resemble our own. These authors focus mainly on those behaviours or actions that the participants suffer on a daily or weekly basis, and even though the prevalence is lower, since the rest of the frequencies are not summed up, in their study it also highlights that they have been under an excessive workload (16%), comprising assigned tasks of an inferior or more unpleasant nature, on occasions as a form of punishment (14%), and being ignored or excluded (11%).

Depending on the methodology employed, different results have been obtained, as observed in another study by Samadzadeh and Aghamohammadi [34], which analyzed the violence that comes from nursing staff or companions, but not from patients and families. Findings indicates that the main reason why students are harassed by nursing staff is the lack of registered professionals in their charge, which causes them to see students as an extra workload. This ends up causing the violent acts that students have suffered the most and is due to the lack of awareness that the instructors have about the functions that the students must not perform, assigning them on occasions tasks that they consider inappropriate according to their level of responsibility (40%).

Despite the fact that the prevalence of this type of violence or abuse is low, both in our study and in the revised literature, it seems to us of special importance to highlight it, due to the physical and psychological consequences that it can have for the person who has been a victim of said abuses. We found a prevalence of threats of violence or physical abuse of 3% (*n* = 12) and a prevalence of 7.5% (*n* = 31) in relation to having suffered intimidating conduct that includes physical space invasion, pushes, etc. This assumes that 43 of our students have suffered physical abuse or intimidating conduct on at least one occasion in the last six months prior to the inquiry. Our prevalence in relation to these attitudes is along the lines of results obtained in another similar study [39], in which there was a differentiation according to the type of physical violence found: being pushed (5%); being hit (2.5%); being slapped (3%); and being injured with an object or weapon (2.8%). In other reviewed studies [9,25,26], the prevalence of physical abuse, without distinguishing the type of abuse, is also low, at 5%. In some investigations, the prevalence of physical abuse is higher, with a finding of between 5 and 10%, and around 16% if victims have been verbally threatened when receiving physical abuse [12,27,34,40].

Of particular note is the study by Karatas et al. [11], in which a prevalence of 30% in physical abuse is found among participants in their study, and the same prevalence in relation to being threatened with suffering physical harm. We also found high prevalences in another study [37], with 18% of physical abuse and 33% of cases of threats involving receiving said abuse.

In our study, we found that 19 participants (4.6%) “sometimes” felt sexually harassed during the performance of their clinical practices. Our results are in line with those found in other revised investigations, in which the prevalence of sexual abuse ranges between 0 and 10% [12,34]. Although it is true that behaviors related to sexual abuse are not the most prevalent suffered by students, they should be highlighted due to the seriousness of such acts and the consequences that may be suffered by those who have been victims.

As a recommendation to practice and for future research it is necessary to discuss the consequences for the victims who have suffered physical and/or sexual harassment due to their own characteristics. These debates should be carried out inside both academic and clinical settings (healthcare organizations), involving nurse practitioners, nursing students, managers, psychologists, and professors. Describing and being aware of the exposure to negative attitudes when bullying behaviours are experienced will further assist researchers and managers in addressing this major problem in the nursing setting. 

Furthermore, although there is quantitative research that indicates the general consequences of suffering bullying, robust qualitative design studies should be conducted to bring new insights in the knowledge and awareness by exploring victims’ experience of bullying in these settings.

As future directions we would like to point out that the reason behind the differences detected in the prevalence of physical abuse against students is unclear. In our understanding of the phenomena, it could be related to cultural, social, or relational factors of the studied population and the setting in which the research was carried out. A more in-depth study would be necessary to provide a more accurate explanation of these findings.

## 5. Conclusions

A high prevalence of bullying among nursing students at the University of Oviedo was detected among young and single female students. Less than a quarter of the participants declared having been harassed during clinical practice. Psychological harassment was found to be the most prevalent factor, with a higher mean score in half of the included students. No associations were found between socio-demographic characteristics and bullying.

## Figures and Tables

**Table 1:** Sample description.

Variable	N (%)
Gender	
Female	349 (84.9)
Male	62 (15.1)
Academic year	
2nd	108 (26.3)
3rd	182 (44.3)
4th	121 (29.4)
Center	
Main	253 (61.6)
Affiliated	158 (38.4)
Marital status	
Single/Divorced	369 (89.8)
Married	42 (10.2)

**Table 2:** Have you been harassed at work?

	N	%
No	339	82.5
Yes, very rarely	24	5.8
Yes, now and then	43	10.5
Yes, several times per month	0	0
Yes, several times per week	3	0.7
Yes, almost daily	2	0.5

**Table 3:** Negative attitudes.

NAQ-R Items		N (%)				
		Never 1	Occasionally 2	Monthly 3	Weekly 4	Daily 5
1	Someone withholding information which affects your performance	255 (62)	137 (33.3)	12 (2.9)	5 (1.2)	2 (0.5)
2	Being humiliated or ridiculed in connection with your work	306 (74.5)	88 (21.4)	10 (2.4)	6 (1.5)	1 (0.2)
3	Being ordered to do work below your level of competence	138 (33.6)	195 (47.4)	26 (6.3)	37 (9)	15 (3.6)
4	Having key areas of responsibility removed or replaced with more trivial or unpleasant tasks	165 (40.1)	168 (40.9)	34 (8.3)	30 (7.3)	14 (3.4)
5	Spreading of gossip and rumors about you	354 (86.1)	48 (11.7)	7 (1.7)	2 (0.5)	0
6	Being ignored or excluded	288 (70.1)	95 (23.1)	11 (2.7)	12 (2.9)	5 (1.2)
7	Having insulted or offensive remarks made about your person, your attitudes or your private life	345 (83.9)	52 (12.7)	10 (2.4)	4 (1.0)	0
8	Being shouted at or being the target of spontaneous anger	297 (72.3)	95 (23.1)	11 (2.7)	6 (1.5)	2 (0.5)
9	Intimidating behaviours such as finger pointing, invasion of personal space, shoving, blocking your way	380 (92.5)	24 (5.8)	5 (1.2)	2 (0.5)	0
10	Hints or signals from others that you should quit your job	334 (81.3)	59 (14.4)	13 (3.2)	3 (0.7)	2 (0.5)
11	Repeated reminders of your errors or mistakes	284 (69.1)	98 (23.8)	12 (2.9)	15 (3.5)	2 (0.5)
12	Being ignored or facing a hostile reaction when you approach	253 (61.6)	125 (30.4)	22 (5.4)	9 (2.2)	2 (0.5)
13	Persistent criticism of your errors or mistakes	324 (78.8)	68 (16.5)	11 (2.7)	7 (1.7)	1 (0.2)
14	Having your opinions ignored	199 (40.1)	167 (40.6)	22 (5.4)	9 (2.2)	14 (3.4)
15	Practical jokes carried out by people you don’t get along with	347 (84.4)	51 (12.4)	10 (2.4)	3 (0.7)	0
16	Being given tasks with unreasonable deadlines	331 (80.5)	62 (15.1)	16 (3.9)	2 (0.5)	0
17	Having allegations made against you	357 (86.9)	41 (10)	12 (2.9)	1 (0.2)	0
18	Excessive monitoring of your work	210 (51.1)	136 (36.1)	34 (8.3)	21 (5.1)	10 (2.4)
19	Pressure not to claim something to which by right you are entitled (e.g., sick leave, holiday)	372 (90.5)	30 (7.3)	6 (1.5)	3 (0.7)	0
20	Being the subject of excessive teasing and sarcasm	359 (87.3)	45 (10.9)	5 (1.2)	2 (0.5)	0
21	Being exposed to an unmanageable workload	226 (55)	118 (28.7)	47 (11.4)	11 (2.7)	8 (1.9)
22	Threats of violence or physical abuse or actual abuse	397 (96.6)	11 (2.7)	1 (0.2)	1 (0.2)	0
23	Feeling sexually harassed in your workplace	391 (95.1)	19 (4.6)	0	0	0

**Table 4:** NAQ-R factors and total score.

		*n*	Mean	SD	Percentiles (%)				
					0	25	50	75	100
Factor	Psychological harassment	411	21.52	6.24	16.00	17.00	20.00	24.00	52.00
	Work-related harassment	410	13.94	4.39	9.00	11.00	13.00	16.00	31.00
	Physical abuse	410	5.68	1.14	5.00	5.00	5.00	6.00	12.00
NAQ-R total score		410	32.76	8.82	24.00	27.00	30.00	36.00	75.00

## Data Availability

The original contributions presented in the study are included in the article material, further inquiries can be directed to the corresponding authors.

## References

[1] Leymann H. (1990). Mobbing and Psychological Terror at Workplaces. Violence Vict..

[2] Einarsen S., Skogstad A. (1996). Bullying at Work: Epidemiological Findings in Public and Private Organizations. Eur. J. Work Organ. Psychol..

[3] Zapf D., Knorz C., Kulla M. (1996). On the Relationship between Mobbing Factors, and Job Content, Social Work Environment, and Health Outcomes. Eur. J. Work Organ. Psychol..

[4] Dellasega C. (2009). Bullying among Nurses. Am. J. Nurs..

[5] Rutherford D., Smith C., Bresler S., Gillespie G. (2020). Emotions and Feelings Evoked in Nursing Students Exposed to Bullying Behaviors in Clinical Settings. J. Nurs. Educ. Pract..

[6] Alshawush K., Hallett N., Radbury-Jones C. (2022). The Impact of Transition Programmes on Workplace Bullying, Violence, Stress and Resilience for Students and New Graduate Nurses: A Scoping Review. J. Clin. Nurs..

[7] Laschinger H.K.S., Grau A.L. (2012). The Influence of Personal Dispositional Factors and Organizational Resources on Workplace Violence, Burnout, and Health Outcomes in New Graduate Nurses: A Cross-Sectional Study. Int. J. Nurs. Stud..

[8] Thomas C.M. (2010). Teaching Nursing Students and Newly Registered Nurses Strategies to Deal with Violent Behaviors in the Professional Practice Environment. J. Contin. Educ. Nurs..

[9] Fernández-Gutiérrez L., Mosteiro-Díaz M.P. (2021). Bullying in Nursing Students: A Integrative Literature Review. Int. J. Ment. Health Nurs..

[10] Al-Qadi M.M. (2021). Workplace Violence in Nursing: A Concept Analysis. J. Occup. Health.

[11] Karatas H., Ozturk C., Bektas M. (2017). A Study of Bullying Against Nursing Students. J. Nurs. Res..

[12] Budden L.M., Birks M., Cant R., Bagley T., Park T. (2017). Australian Nursing Students’ Experience of Bullying and/or Harassment during Clinical Placement. Collegian.

[13] Hakojärvi H.R., Salminen L., Suhonen R. (2014). Health Care Students’ Personal Experiences and Coping with Bullying in Clinical Training. Nurse Educ. Today.

[14] Amoo S., Menlah A., Garti I., Appiah E. (2021). Bullying in the Clinical Setting: Lived Experiences of Nursing Students in the Central Region of Ghana. PLoS ONE.

[15] Fasanya B., Dada E. (2016). Workplace Violence and Safety Issues in Long-Term Medical Care Facilities: Nurses’ Perspectives. Saf. Health Work.

[16] Fernández-Fernández J., Sánchez-Valdeón L., Casado-Verdejo I., Gómez-Salgado J., Méndez-Martínez C., García-Suárez M., Fernández-García D. (2022). Análisis de La Intimidación y Acoso Experimentado Por Estudiantes de Enfermería de Cuarto Curso Durante Sus Prácticas Clínicas [Analysis of Bullying and Harassment Experienced by Fourth-Year Nursing Students in Their Clinical Practice]. Rev. Esp. Salud Publica.

[17] Kivimäki M., Elovainio M., Vahtera J. (2000). Workplace Bullying and Sickness Absence in Hospital Staff. Occup. Environ. Med..

[18] McKenna B., Smith N., Poole S., Coverdale J. (2003). Horizontal Violence: Experiences of Registered Nurses in Their First Year of Practice. J. Adv. Nurs..

[19] Bambi S., Becattini G., Giusti G., Mezzetti A., Guazzini A., Lumini E. (2014). Lateral Hostilities among Nurses Employed in Intensive Care Units, Emergency Departments, Operating Rooms, and Emergency Medical Services. A National Survey in Italy. Dimens. Crit. Care Nurs..

[20] Saraiva D., Pinto A. (2011). Mobbing in a Nursing Context. Rev. Enferm. Ref..

[21] von Elm E., Altman D., Egger M., Pocock S., Gotzsche P., Vandenbroucke J. (2007). The Strengthening the Reporting of Observational Studies in Epidemiology (STROBE) Statement: Guidelines for Reporting Observational Studies. Lancet.

[22] Einarsen S., Hoel H., Notelaers G. (2009). Measuring Exposure to Bullying and Harassment at Work: Validity, Factor Structure and Psychometric Properties of the Negative Acts Questionnaire-Revised. Work Stress.

[23] González-Trijueque D., Graña Gómez J. (2013). Adaptación psicométrica de una versión española del cuestionario de conductas negativas revisado (NAQ-R). Psicopatol. Clínica Leg. Forense.

[24] Instituto Nacional de Estadística (2020). Profesionales Sanitarios Colegiados por Sexo. Series Desde 1952.

[25] Palaz S. (2013). Turkish Nursing Students’ Perceptions and Experiences of Bullying Behaviors in Nursing Education. J. Nurs. Educ. Pract..

[26] Reem-Mabrouk A.E.R. (2013). Student Nurses’ Bullying Behaviors and Coping Strategies Used in Clinical Settings.

[27] Clarke C.M., Kane D., Rajacich D.L., Lafreniere K.D. (2012). Bullying in Undergraduate Clinical Nursing Education. J. Nurs. Educ..

[28] Cooper J., Walker J., Asket R., Robinson J., McNair M. (2011). Student’s Perceptions of Bullying Behaviors by Nursing Faculty. Issues Educ. Res..

[29] Smith C., Gillespie G., Brown K., Grubb P.L. (2016). Seeing Students Squirm: Nursing Students’ Experiences of Bullying Behaviors during Clinical Rotations. J. Nurs. Educ..

[30] Minton C., Birks M. (2019). “You Can’t Escape It”: Bullying Experiences of New Zealand Nursing Students on Clinical Placement. Nurse Educ. Today.

[31] Khamis Mohamed L. (2019). Experiences of Saudi Female Students towards the Phe-Nomena of Bullying Behaviors during Nursing Education Program. Am. J. Nurs. Res..

[32] Moreno-Cubillos C., Spúlveda-Gallego L. (2013). Violencia y Discriminación Contra Estudiantes de Enfermería En Una Universidad Pública Colombiana. Investig. Educ. Enfermería.

[33] Ren L., Kim H. (2017). Effects of Bullying Experience on Psychological Well-Being Mediated by Conflict Management Styles and Psychological Empowerment among Nursing Students in Clinical Placement: A Structural Equation Modeling Approach. J. Korean Acad. Nurs..

[34] Samadzadeh S., Aghamohammadi M. (2018). Violence against Nursing Students in the Workplace: An Iranian Experience. Int. J. Nurs. Educ. Scholarsh..

[35] Curtis J., Bowen I., Reid A. (2007). You Have No Credibility: Nursing Students’ Experiences of Horizontal Violence. Nurse Educ. Pract..

[36] Stevenson K., Randle J., Grayling I. (2006). Inter-Group Conflict in Health Care: UK Students’ Experiences of Bullying and the Need for Organisational Solutions. Online J. Issues Nurs..

[37] Abdelaziz E., Abu-Snieneh H. (2021). The Impact of Bullying on the Mental Health and Academic Achievement of Nursing Students. Perspect. Psychiatr. Care.

[38] Randle J. (2003). Bullying in the Nursing Profession. J. Adv. Nurs..

[39] Tee S., Üzar Özçetin Y., Russell-Westhead M. (2016). Workplace Violence Experi-Enced by Nursing Students: A UK Survey. Nurse Educ. Today.

[40] Prasad K. (2014). Bullying from Nursing: Students’ Perspective. Nurs. J. India.

